# Association of Genetically Determined Aldehyde Dehydrogenase 2 Activity with Diabetic Complications in Relation to Alcohol Consumption in Japanese Patients with Type 2 Diabetes Mellitus: The Fukuoka Diabetes Registry

**DOI:** 10.1371/journal.pone.0143288

**Published:** 2015-11-23

**Authors:** Yasuhiro Idewaki, Masanori Iwase, Hiroki Fujii, Toshiaki Ohkuma, Hitoshi Ide, Shinako Kaizu, Tamaki Jodai, Yohei Kikuchi, Atsushi Hirano, Udai Nakamura, Michiaki Kubo, Takanari Kitazono

**Affiliations:** 1 Department of Medicine and Clinical Science, Graduate School of Medical Sciences, Kyushu University, Fukuoka, Japan; 2 Diabetes Center, Hakujyuji Hospital, Fukuoka, Japan; 3 Division of General Internal Medicine, School of Oral Health Science, Kyushu Dental University, Kitakyushu, Japan; 4 Division of Research Management, Center for Cohort Studies, Graduate School of Medical Sciences, Kyushu University, Fukuoka, Japan; 5 Center for Integrative Medical Sciences, RIKEN, Yokohama, Japan; National Cancer Center, JAPAN

## Abstract

Aldehyde dehydrogenase 2 (ALDH2) detoxifies aldehyde produced during ethanol metabolism and oxidative stress. A genetic defect in this enzyme is common in East Asians and determines alcohol consumption behaviors. We investigated the impact of genetically determined ALDH2 activity on diabetic microvascular and macrovascular complications in relation to drinking habits in Japanese patients with type 2 diabetes mellitus. An *ALDH2* single-nucleotide polymorphism (rs671) was genotyped in 4,400 patients. Additionally, the relationship of clinical characteristics with ALDH2 activity (ALDH2 *1/*1 active enzyme activity vs. *1/*2 or *2/*2 inactive enzyme activity) and drinking habits (lifetime abstainers vs. former or current drinkers) was investigated cross-sectionally (n = 691 in *1/*1 abstainers, n = 1,315 in abstainers with *2, n = 1,711 in *1/*1 drinkers, n = 683 in drinkers with *2). The multiple logistic regression analysis for diabetic complications was adjusted for age, sex, current smoking habits, leisure-time physical activity, depressive symptoms, diabetes duration, body mass index, hemoglobin A_1c_, insulin use, high-density lipoprotein cholesterol, systolic blood pressure and renin-angiotensin system inhibitors use. Albuminuria prevalence was significantly lower in the drinkers with *2 than that of other groups (odds ratio [95% confidence interval (CI)]: *1/*1 abstainers as the referent, 0.94 [0.76–1.16] in abstainers with *2, 1.00 [0.80–1.26] in *1/*1 drinkers, 0.71 [0.54–0.93] in drinkers with *2). Retinal photocoagulation prevalence was also lower in drinkers with ALDH2 *2 than that of other groups. In contrast, myocardial infarction was significantly increased in ALDH2 *2 carriers compared with that in ALDH2 *1/*1 abstainers (odds ratio [95% CI]: *1/*1 abstainers as the referent, 2.63 [1.28–6.13] in abstainers with *2, 1.89 [0.89–4.51] in *1/*1 drinkers, 2.35 [1.06–5.79] in drinkers with *2). In summary, patients with type 2 diabetes and ALDH2 *2 displayed a lower microvascular complication prevalence associated with alcohol consumption but a higher macrovascular complication prevalence irrespective of alcohol consumption.

## Introduction

As alcoholic beverages are used in social and ritual settings in many cultures, the impact of alcohol consumption on health and disease is inevitable. According to the World Health Organization, the greater the economic wealth of a country, the more alcohol is consumed. Furthermore, the highest increase in alcohol consumption is expected in the populations of the western pacific region including East Asia [1]. As economic growth is typically accompanied by a type 2 diabetes mellitus epidemic, increased alcohol consumption is similarly expected to be associated with the diabetes epidemic in East Asia. There are many studies investigating the effect of alcohol consumption on cardiovascular disease in diabetic and nondiabetic populations. The American Diabetes Association indicates in their “Nutrition therapy recommendations for the management of adults with diabetes” that moderate alcohol consumption may confer cardiovascular risk reduction and mortality benefits in people with diabetes, as in the general population [2]. However, they do not comment on the effect of alcohol consumption on microvascular complications.

Acetaldehyde dehydrogenase 2 (ALDH2) is a key enzyme involved in alcohol metabolism that detoxifies acetaldehyde into acetic acid. The *ALDH2* gene has a G-to-A missense mutation (rs671) in which glutamate at position 504 is replaced by lysine, named *1 and *2, respectively. The ALDH2 *2 allele causes almost complete loss of enzyme activity [3], and heterozygous individuals (ALDH2 *1/*2) display 1/16 (6.3%) of the wild-type enzymatic activity, because ALDH2 functions as a tetramer. Carriers of the ALDH2 *2 allele, which is more common in East Asians (30–50%) than in Caucasians (lower than 5%), display an “alcohol flushing” phenomenon. These individuals present with a headache and palpitation, even after consuming only a small amount of alcohol. Therefore, alcohol consumption is largely determined by the functional *ALDH2* variant rs671 in Japanese people [4].

In addition to the impact on drinking behaviors, ALDH2 has attracted considerable attention recently because of its anti-oxidative properties [5–8]. ALDH2 may play an important role in oxidizing endogenous aldehydes, such as 4-hydroxy-2-nonenal and malondialdehyde, produced by oxidative stress. ALDH2-deficient rodents displayed myocardial or brain ischemia exacerbation, and ALDH2 activation conferred cardio- and neuro-protective properties. Thus, ALDH2 activators may display novel therapeutic potential. Furthermore, a genome-wide association study demonstrated that an *ALDH2* single-nucleotide polymorphism (SNP) (rs671) was strongly associated with coronary artery disease in a Japanese population [9]. In addition, diabetic retinopathy development may be associated with the *ALDH2* SNP in Japanese patients with type 2 diabetes mellitus [10, 11]. These findings suggest that the *ALDH2* SNP may affect diabetic complication development, not only through alcohol consumption but also through mechanisms independent of alcohol consumption. Thus, we designed the present study to investigate the impact of genetically determined ALDH2 activity on diabetic microvascular and macrovascular complications in relation to drinking habits in Japanese patients with type 2 diabetes mellitus.

## Methods

### Study subjects

The Fukuoka Diabetes Registry is a multicenter prospective study investigating the influence of modern treatments on prognosis in patients with diabetes mellitus regularly attending teaching hospitals certified by the Japan Diabetes Society or certified diabetologists’ clinics in Fukuoka Prefecture, Japan [UMIN Clinical Trial Registry 000002627) [12]. A total of 5,131 diabetic patients aged ≥20 years were registered between April 2008 and October 2010. Exclusion criteria were: (1) patients with drug-induced diabetes or receiving corticosteroid treatment; (2) patients undergoing renal replacement therapy; (3) patients with serious diseases other than diabetes, such as advanced malignancy or decompensated liver cirrhosis; and (4) patients unable to visit a diabetologist regularly. Patients with type 1 diabetes mellitus (negative serum C-peptide and/or positive anti-glutamic acid decarboxylase antibody) and those who had already eaten breakfast were excluded. In total, 4,400 individuals (2,483 men and 1,917 women) were enrolled in this cross-sectional study. The study was conducted with the approval of the Kyushu University Institutional Review Board, and written informed consent was obtained from all participants.

### Clinical evaluations

Participants completed a self-administered questionnaire to collect information on diabetes duration, smoking habits, physical activity, past medical history including retinal photocoagulation, dysesthesia of both feet, myocardial infarction, brain infarction and malignant neoplasm. Participants reported items on the frequency of their habitual alcohol intake and the kinds and amounts of alcoholic beverages customarily consumed [13]. These measurements were converted into daily alcohol intake (g/d). Former drinkers were defined as those who previously consumed alcohol regularly but no longer did at the time of evaluation. Body mass index (BMI) was calculated using the height and weight of each subject, and obesity was defined according to the Japan Society for the Study of Obesity thresholds (BMI ≥25 kg/m^2^). Waist circumference was measured at the umbilical level, with the subject in the standing position, by a trained staff member. Blood pressure was measured with the subject in a seated position. Subjects’ medical records were reviewed for all medications including antihypertensive drugs and lipid-lowering drugs. Hypertension was defined as systolic blood pressure ≥140 mmHg, diastolic blood pressure ≥90 mmHg and/or taking blood pressure-lowering drugs. Leisure-time physical activity information was obtained using a self-reported questionnaire, and metabolic equivalent (met) hours per week was calculated using Ainsworth’s methods [14, 15]. The presence of depressive symptoms was assessed using the Center for Epidemiologic Studies Depression Scale with 16 as a cutoff score [16, 17].

### Laboratory measurements

Blood was collected by venipuncture following an overnight fast, and spot urine samples were obtained. The *ALDH2* SNP (rs671) was genotyped using the multiplex polymerase chain reaction (PCR)-based Invader assay (Third Wave Technologies, Madison, WI) [18]. HbA_1c_ was determined using high-performance liquid chromatography (Tosoh Corp., Tokyo, Japan), plasma glucose by the glucose oxidase method, serum C-peptide by chemiluminescent immunoassay (Kyowa Medex, Tokyo, Japan; Siemens Healthcare Diagnostics, Tokyo, Japan), serum high-sensitivity C-reactive protein (HS-CRP) by latex immunonephelometry (Siemens Healthcare Diagnostics, Tokyo, Japan), urinary albumin by immunonephelometry (Medical and Biological Laboratories, Nagoya, Japan) and serum total cholesterol, low-density lipoprotein (LDL) cholesterol, high-density lipoprotein (HDL) cholesterol, triglyceride, creatinine and urine creatinine by enzymatic methods. The estimated glomerular filtration rate (eGFR) was calculated using the equation proposed by the Japanese Society of Nephrology [19]. Albuminuria was defined as urinary albumin excretion ≥30 mg/g creatinine, and chronic kidney disease (CKD) was defined as eGFR <60 ml/min/1.73 m^2^. β-cell function and insulin resistance were estimated based on fasting glucose and C-peptide concentrations using the homeostasis model assessment (HOMA) calculator, version 2.2.2 [20], and expressed as HOMA β-cell function (HOMA2%-B) and HOMA-insulin resistance (HOMA2-IR), respectively.

### Statistical analysis

Triglyceride and HS-CRP levels were log-transformed for the statistical analyses because both displayed a skewed distribution. A one-way analysis of variance test was used to analyze differences between group means and the Tukey-Kramer test was used to perform multiple comparisons of group means. Categorical variable comparisons were performed using Pearson’s chi-square test. Age- and sex-adjustments were performed by multiple regression analysis. The multivariate-adjusted odds ratio (OR) and 95% confidence interval (CI) for albuminuria, CKD, photocoagulation, myocardial infarction, brain infarction and malignant neoplasm were calculated using a multiple logistic regression model. Multivariate adjustments included age, sex, current smoking habits, leisure-time physical activity, depressive symptoms, diabetes duration, BMI, HbA_1c_, insulin use, HDL cholesterol, systolic blood pressure and renin-angiotensin system inhibitor use. The interaction between genetically determined ALDH2 activity and drinking status with respect to diabetic complications was assessed by adding an interaction term to the statistical model. All analyses were performed using the JMP version 11 (SAS Institute Inc., Cary, NC, USA). P-values <0.05 were considered to be statistically significant in all analyses.

## Results

Among the 4,400 participants, 2,402 (54.6%), 1,655 (37.6%) and 343 (7.8%) were homozygous for the *1 allele, heterozygous and homozygous for *2 allele, respectively, at the *ALDH2* rs671 locus. The observed genotype frequency distribution was consistent with the Hardy-Weinberg equilibrium. The prevalence of lifetime abstainers, former drinkers and current drinkers was 691 (28.8%), 377 (15.7%) and 1,334 (55.5%) in ALDH2 *1/*1 carriers, 993 (60.0%), 280 (16.9%) and 382 (23.1%) in ALDH2 *1/*2 carriers, and 322 (93.9%), 12 (3.5%) and 9 (2.6%) in ALDH2 *2/*2 carriers, respectively. As there were relatively few former and current drinkers among ALDH2 *2 carriers, we combined ALDH2 *1/*2 and *2/*2 carriers into one group (ALDH2 *2 carriers, inactive enzyme group) for further analyses.

As displayed in Table 1, there were 691 (28.8%) lifetime abstainers and 1,711 (71.2%) former or current drinkers with ALDH2 *1/*1 (active enzyme activity). In contrast, there were 1,315 (65.8%) lifetime abstainers and 683 (34.2%) former or current drinkers with the ALDH2 *2 allele (inactive enzyme activity). Age did not significantly differ between the groups, but there were more male drinkers than female drinkers. Of those who consumed alcohol, the proportion of current drinkers was significantly lower in ALDH2 *2 carriers than that in ALDH2 *1/*1 carriers. Current alcohol consumption was significantly lower in those with the ALDH2 *2 allele than in those with ALDH2 *1/*1 allele. The proportion of light drinkers (<10 g/day) was significantly higher in ALDH2 *2 carriers than that in ALDH2 *1/*1 carriers. In contrast, the proportion of heavy drinkers (≥40 g/day) was lower in ALDH2 *2 carriers than that in ALDH2 *1/*1 carriers. Alcohol consumption duration was shorter in ALDH2 *2 carriers than that in ALDH2 *1/*1 carriers. The proportion of current smokers and leisure-time physical activity were both higher in those that consumed alcohol than in the lifetime abstainers. Furthermore, the prevalence of depressive symptoms significantly differed between the groups; however, these significance was attenuated or lost after adjusting for age and sex.

**Table 1:** Clinical characteristics according to ALDH2 activity (ALDH2 *1/*1 active vs. *1/*2 or *2/*2 inactive) and drinking status in Japanese patients with type 2 diabetes.

	Lifetime abstainers		Former or current drinkers		p	
*ALDH2* rs671	*1/*1	*1/*2 or *2 /*2	*1/*1	*1/*2 or *2 /*2	Unadjusted	Age- and sex-adjusted
n	691	1315	1711	683	-	-
Age (years)	65.0±12.0	65.6±10.6	64.8±9.9	65.2±10.1	ns	ns
Sex (male %)	9.4%	39.7%	76.3%	86.5%	<0.0001	<0.0001
Current drinker (%)	-	-	78.0%	57.3%^a^	<0.0001	<0.0001
Current alcohol consumption (g/day)	-	-	26.3±29.9	18.4±25.9^a^	<0.0001	<0.0001
<10 g/day	-	-	38.1%	51.2%	<0.0001	<0.0001
10–40 g/day	-	-	39.7%	35.8%		
≥40 g/day	-	-	22.2%	13.0%		
Duration of alcohol consumption (years)	-	-	40.4±12.6	36.3±13.6^a^	<0.0001	<0.0001
Current smoker (%)	6.8%	14.5%	22.2%	26.2%	<0.0001	<0.05
Leisure-time physical activity (met・hr/week)	9.5±12.6	10.5±14.3	13.0±16.1^b^ ^c^	13.5±14.8^b^ ^d^	<0.0001	ns
Depressive symptoms (%)	11.6%	8.8%	9.3%	6.4%	<0.05	ns

Values are mean±SD.

^b^p<0.001 vs. lifetime abstainers with *1/*1,

^d^p<0.01,

^c^p<0.001 vs. lifetime abstainers with *2,

^a^p<0.001 vs. drinkers with *1/*1 in an unadjusted model.

Metabolic parameters according to ALDH2 activity and drinking status are displayed in Table 2. BMI was moderately lower in individuals that consumed alcohol than that in the lifetime abstainers. Furthermore, obesity prevalence was lower in those that consumed alcohol. Waist circumference did not differ between the groups. Diabetes duration was moderately longer in the drinkers than that in the lifetime abstainers, although the significance was lost following adjustment for age and sex. Fasting plasma glucose levels did not differ between the groups, whereas HbA_1c_ was significantly lower in the drinkers than that in lifetime abstainers. Fasting serum C-peptide, HOMA2%-B, HOMA2-IR and HS-CRP did not differ between the groups. Similarly, the proportion of participants treated with oral hypoglycemic agents did not differ between the groups. The proportion of participants treated with insulin was lower in the drinking group than that in the lifetime abstainers; however, the difference was no longer significant following age and sex adjustment. Total cholesterol levels significantly differed between the groups only in an unadjusted model. LDL cholesterol levels tended to be lower in the drinkers than those in the lifetime abstainers. HDL cholesterol levels were significantly higher in the drinkers than those in the lifetime abstainers after adjustment for age and sex (age- and sex-adjusted mean (±SEM) 1.46±0.02 mmol /l in lifetime abstainers with ALDH2 *1/*1, 1.43±0.01 mmol/l in lifetime abstainers with ALDH2 *2, 1.57±0.01 mmol/l in drinkers with ALDH2 *1/*1, 1.50±0.02 mmol/l in drinkers with ALDH2 *2). Non-HDL cholesterol levels tended to be lower in the drinkers than those in the lifetime abstainers. Triglyceride levels did not differed between the groups. The proportion of participants treated with statins was lower in the drinkers than that in the lifetime abstainers.

**Table 2:** Metabolic parameters according to ALDH2 activity (ALDH2 *1/*1 active vs. *1/*2 or *2/*2 inactive) and drinking status in Japanese patients with type 2 diabetes.

	Lifetime abstainers		Former or current drinkers		p	
*ALDH2* rs671	*1/*1	*1/*2 or *2 /*2	*1/*1	*1/*2 or *2 /*2	Unadjusted	Age-and sex-adjusted
BMI (kg/m^2^)	24.4±5.0	23.9±3.8^a^	23.6±3.4^b^	23.4±3.2^b^	<0.0001	<0.001
Obesity (BMI≥25.0) (%)	36.8%	33.3%	28.2%	26.2%	<0.0001	<0.01
Waist circumstances (cm)	86.4±12.3	85.7±10.6	85.5±9.4	85.1±9.1	ns	ns
Duration of diabetes (years)	14.3±9.6	15.1±10.4	16.4±10.7^b^ ^c^	16.0±10.7^a^	<0.0001	ns
Fasting plasma glucose (mmol/l)	7.7±2.2	7.8±2.2	7.8±2.3	7.7±2.2	ns	ns
HbA_1c_ (%)	7.54±1.08	7.55±1.07	7.31±1.00^b^ ^d^	7.37±0.97^c^ ^e^	<0.0001	<0.0001
HbA_1c_ (mmol/mol)	59±12	59±12	56±11^b^ ^d^	57±11^c^ ^e^	<0.0001	<0.0001
Fasting serum C-peptide (nmol/l)	0.38±0.23	0.39±0.24	0.40±0.22	0.39±0.22	ns	ns
HOMA2%-B	46.5±25.1	45.9±24.4	45.3±22.6	45.8±25.8	ns	ns
HOMA2-IR	1.11±0.60	1.11±0.54	1.13±0.61	1.13±0.66	ns	ns
HS-CRP (mg/l)	0.50 [0.45–0.55]	0.50 [0.46–0.53]	0.48 [0.45–0.51]	0.49 [0.44–0.55]	ns	ns
Oral hypoglycemic agents use (%)	65.4%	63.5%	62.4%	60.0%	ns	ns
Insulin use (%)	30.0%	31.1%	26.6%	26.5%	<0.05	ns
Total cholesterol (mmol/l)	5.17±0.83	5.07±0.86^a^	4.97±0.83^b^ ^f^	4.91±0.78^b^ ^d^	<0.0001	ns
LDL cholesterol (mmol/l)	2.96±0.70	2.96±0.70	2.78±0.70^b^ ^d^	2.86±0.68^a^ ^c^	<0.0001	<0.0001
HDL cholesterol (mmol/l)	1.56±0.39	1.46±0.36^b^	1.51±0.42^d^	1.43±0.39^b^ ^g^	<0.0001	<0.0001
non-HDL cholesterol (mmol/l)	3.61±0.78	3.61±0.81	3.46±0.78^b^ ^d^	3.48±0.73^c^ ^e^	<0.0001	<0.01
Triglyceride (mmol/l)	1.17 [1.13–1.22]	1.21 [1.17–1.24]	1.22 [1.20–1.25]	1.20 [1.15–1.24]	ns	ns
Statin use (%)	52.2%	48.0%	36.4%	39.1%	<0.0001	<0.05

HOMA2%-B, homeostasis model assessment β-cell function; HOMA2-IR, homeostasis model assessment insulin resistance; HS-CRP, high-sensitivity C-reactive protein. Values are expressed as mean ± SD or percentage. HS-CRP and triglyceride are presented as geometric means [95% confidence interval].

^a^p<0.05,

^e^p<0.01,

^b^p<0.001 vs. lifetime abstainers with *1/*1,

^f^p<0.05,

^c^p<0.01,

^d^p<0.001 vs. lifetime abstainers with *2,

^g^p<0.001 vs. drinkers with *1/*1 in an unadjusted model.

Systolic blood pressure did not differ between the groups (Table 3). Although diastolic blood pressure tended to be increased in the drinking group, significance was lost following adjustment for age and sex. The proportion of participants treated with antihypertensive drugs, including renin-angiotensin system inhibitors, calcium channel blockers and diuretics, was significantly lower in drinkers with ALDH2 *2 than that in other groups. Hypertension prevalence, urinary albumin excretion and albuminuria (≥30 mg/gCr) prevalence were also significantly lower in drinkers with the ALDH2 *2 allele compared with that of other groups. However, macroalbuminuria (≥300 mg/gCr) prevalence, eGFR and CKD prevalence did not differ between the groups. Although the prevalence of dysesthesia of both feet did not significantly differ between the groups, the proportion of participants treated with retinal photocoagulation was lower in the drinkers with ALDH2 *2 than that in other groups. Regarding diabetic macroangiopathy, myocardial and brain infarction prevalence was lower in the lifetime abstainers with ALDH2 *1/*1 than that in other groups. The proportion of participants previously diagnosed with malignant neoplasm did not significantly differ between the groups.

**Table 3:** Blood pressure and diabetic complications according to ALDH2 activity (ALDH2 *1/*1 active vs. *1/*2 or *2/*2 inactive) and drinking status in Japanese patients with type 2 diabetes.

	Lifetime abstainers		Former or current drinkers		p	
*ALDH2* rs671	*1/*1	*1/*2 or *2 /*2	*1/*1	*1/*2 or *2 /*2	Unadjusted	Age-and sex-adjusted
Systolic blood pressure (mmHg)	132±18	130±18	130±17	130±16	ns	ns
Diastolic blood pressure (mmHg)	73±11	74±11	75±10^a^ ^b^	75±10^c^	<0.0001	ns
Antihypertensive drug use (%)	57.5%	53.3%	55.8%	46.4%	<0.0001	<0.0001
Renin-angiotensin system inhibitor use (%)	45.3%	43.2%	46.8%	39.1%	<0.01	<0.01
Calcium channel blocker use (%)	36.6%	31.3%	35.9%	26.7%	<0.0001	<0.0001
Diuretics use (%)	12.2%	10.3%	10.6%	7.2%	<0.05	<0.05
Hypertension (%)	67.9%	62.1%	64.1%	57.0%	<0.001	<0.001
Urinary albumin excretion (mg/gCr)	29 [26–33]	29 [27–32]	29 [26–31]	22 [19–25]^b^ ^d^ ^e^	<0.01	<0.001
Albuminuria (≥30 mg/gCr) (%)	38.5%	38.8%	40.5%	33.4%	<0.05	<0.01
Macroalbuminuria (≥300 mg/gCr) (%)	10.6%	11.0%	12.1%	9.8%	ns	ns
eGFR (ml/min/1.73 m^2^)	77±23	75±22	75±22	75±20	<0.05	ns
CKD (%)	18.4%	22.7%	21.2%	21.8%	ns	ns
Retinal Photocoagulation (%)	23.6%	22.7%	22.0%	16.4%	<0.01	<0.05
Dysesthesia of both feet (%)	20.3%	18.9%	20.7%	17.3%	ns	ns
Myocardial infarction (%)	1.2%	4.6%	4.2%	5.7%	<0.0001	<0.05
Brain infarction (%)	4.6%	8.1%	10.1%	9.4%	<0.001	<0.05
Malignant neoplasm (%)	8.5%	7.9%	9.6%	11.6%	ns	ns

eGFR, estimated glomerular filtration rate; CKD, chronic kidney disease. Values are expressed as mean ± SD or percentage. Urinary albumin excretion is presented as geometric means [95% confidence interval].

^c^p<0.05,

^d^p<0.01,

^a^p<0.001 vs. lifetime abstainers with *1/*1,

^b^p<0.01 vs. lifetime abstainers with *2,

^e^p<0.01 vs. drinkers with *1/*1 in an unadjusted model.

Table 4 displays the results of the multiple logistic regression analysis for diabetic complications based on ALDH2 activity and drinking status after adjusting for confounders. Confounders included age, sex, current smoking habits, leisure-time physical activity, depressive symptoms, diabetes duration, BMI, HbA_1c_, insulin use, HDL cholesterol, systolic blood pressure and renin-angiotensin system inhibitors use. Albuminuria prevalence was significantly reduced in the drinkers with the ALDH2 *2 allele compared with that of other groups (OR 0.71 [95% CI 0.54–0.93], p<0.05 vs. lifetime abstainers with ALDH2 *1/*1 as the referent; OR 0.76 [0.61–0.95], p<0.05 vs. lifetime abstainers with ALDH2 *2 as the referent; OR 0.71 [0.58–0.87], p<0.001 vs. drinkers with ALDH2 *1/*1 as the referent). A significant interaction was observed between ALDH2 activity and drinking status with respect to albuminuria in an age- and sex-adjusted model (p<0.05) but not in a multivariate-adjusted model (p = 0.059). CKD prevalence did not differ between the groups. Retinal photocoagulation prevalence was reduced in the drinkers with ALDH2 *2 compared with that in other groups (OR 0.69 [0.50–0.95], p<0.05 vs. lifetime abstainers with ALDH2 *1/*1 as the referent; OR 0.74 [0.56–0.97], p<0.05 vs. lifetime abstainers with ALDH2 *2 as the referent; OR 0.71 [0.55–0.91], p<0.01 vs. drinkers with ALDH2 *1/*1 as the referent). A significant interaction was observed between ALDH2 activity and drinking status with respect to retinal photocoagulation in an age- and sex-adjusted model (p<0.05) but not in a multivariate-adjusted model (p = 0.10). In contrast, myocardial and brain infarctions were significantly increased in ALDH2 *2 carriers compared with that in the lifetime abstainers with ALDH2 *1/*1, irrespective of drinking status. Brain infarction prevalence was significantly increased in the drinkers compared with that in the lifetime abstainers among ALDH2 *1/*1 carriers. Malignant neoplasm prevalence was significantly increased in drinkers compared with that in lifetime abstainers among ALDH2 *2 carriers (OR 1.54 [1.10–2.15], p<0.05 vs. lifetime abstainers with ALDH2 *2 as the referent).

**Table 4:** Multiple logistic regression analysis for diabetic complications according to ALDH2 activity (ALDH2 *1/*1 active vs. *1/*2 or *2/*2 inactive) and drinking status in Japanese patients with type 2 diabetes.

	Lifetime abstainers		Former or current drinkers	
*ALDH2* rs671	*1/*1	*1/*2 or *2 /*2	*1/*1	*1/*2 or *2 /*2
Albuminuria (≥30 mg/gCr)	Referent	0.94 [0.76–1.16]	1.00 [0.80–1.26]	0.71 [0.54–0.93]^a^
CKD	Referent	1.16 [0.89–1.51]	1.03 [0.78–1.38]	1.06 [0.76–1.47]
Photocoagulation	Referent	0.95 [0.74–1.21]	0.98 [0.76–1.28]	0.69 [0.50–0.95]^a^
Myocardial infarction	Referent	2.63 [1.28–6.13]^b^	1.89 [0.89–4.51]	2.35 [1.06–5.79]^a^
Brain infarction	Referent	1.69 [1.12–2.62]^a^	2.16 [1.40–3.40]^c^	1.92 [1.17–3.19]^a^
Malignant neoplasm	Referent	0.91 [0.64–1.29]	1.14 [0.79–1.64]	1.39 [0.92–2.11]

Data represent the multivariate-adjusted OR with 95% confidence interval.

^a^p<0.05,

^b^p<0.01,

^c^p<0.001 vs lifetime abstainers with *1/*1. Multivariate adjustments include age, sex, current smoking habits, leisure-time physical activity, depressive symptoms, diabetes duration, BMI, HbA_1c_, insulin use, HDL cholesterol, systolic blood pressure and renin-angiotensin system inhibitor use.

## Discussion

The present study demonstrated that drinkers with the ALDH2 *2 allele generally consumed light to moderate amounts of alcohol. Furthermore, this group had a lower prevalence of albuminuria and retinal photocoagulation compared with that of other groups, independent of glycemic and blood pressure control, renin-angiotensin system inhibitors use and life style factors. In contrast, myocardial and brain infarction prevalence was significantly increased in ALDH2 *2 carriers compared with that in the lifetime abstainers with ALDH2 *1/*1, irrespective of drinking status. This study is the first to investigate the association of genetically determined ALDH2 activity and diabetic complications in relation to alcohol consumption in a large population of patients with type 2 diabetes mellitus.

Although the effect of the *ALDH2* SNP on diabetic nephropathy development has not been reported, the association of alcohol consumption and CKD has been studied on an epidemiological basis. One Japanese 10-year follow-up study (n = 123,764) observed that moderate (<20 g/day) but not heavy alcohol consumption decreased proteinuria development in a community-based population [21]. In Western populations, two cross-sectional studies in patients with type 1 diabetes demonstrated that the prevalence of overt nephropathy was associated with alcohol consumption, in a U-shaped fashion in one study [22] and in a L-shaped fashion in the other [23], One prospective study in patients with type 2 diabetes mellitus indicated that moderate but not heavy alcohol consumption prevented nephropathy progression during a 5.5-year follow-up (nondrinkers as the referent, moderate drinkers, OR 0.79; heavy drinkers, OR 0.85) [24]. Therefore, the reduced albuminuria prevalence in the drinkers with the ALDH2 *2 allele is consistent with the reported renoprotective action of moderate alcohol consumption.

The reduced prevalence of hypertension in drinkers with the ALDH2 *2 allele may contribute to reduced albuminuria. In a meta-analysis by Chen et al. [25], systolic blood pressure was 7.4 mmHg higher in ALDH2 *1/*1 carriers than that in ALDH2 *2/*2 carriers. Additionally, the risk for hypertension among ALDH2 *1/*1 carriers was 2.5-fold greater than that in ALDH2 *2/*2 carriers. Furthermore, it was reported that, in regular drinkers, systolic blood pressure was lower in ALDH2 *1/*2 carriers than that in ALDH2 *1/*1 carriers in a large-scale validation study of the general Japanese population [26]. Moreover, hypertension prevalence was lower in ALDH2 *2 carriers than that in ALDH2 *1/*1 carriers in drinkers, but not in nondrinkers, in a Chinese population [27]. Although the mechanism by which the ALDH2 *2 allele protects against hypertension in drinkers remains to be elucidated, the direct and indirect vasodilating actions of acetaldehyde may partly explain this effect [27].

Conflicting results have been reported regarding the association of diabetic retinopathy with the *ALDH*2 SNP and alcohol consumption. One cross-sectional study (n = 212) demonstrated that proliferative retinopathy prevalence was significantly lower in ALDH2 *2 allele carriers than that in ALDH2 *1/*1 carriers (2.2% vs. 9.2% p<0.05) [10]. In contrast, a retrospective study (n = 234) revealed that, among drinkers, ALDH2 *2 carriers had a higher incidence of retinopathy than that in ALDH2 *1/*1 carriers [11]. Alcohol consumption was not associated with retinopathy progression in patients with type 2 diabetes mellitus during the 5.5-yearsfollow-up [28]. In contrast, two cross-sectional studies indicated that, in patients with type 1 diabetes mellitus, the proliferative retinopathy prevalence was associated with alcohol consumption, in a U-shaped fashion in one study [22] and in an L-shaped fashion in the other [23]. The mechanisms underlying the beneficial effects of the ALDH2 *2 allele in individuals that consume alcohol remain to be elucidated.

The association of the ALDH2 *2 allele with myocardial infarction has been reported in Japanese [29], Koreans [30] and Chinese [31] populations. The ALDH2 *2 allele was associated with the strongest risk of coronary heart disease in a Japanese genome-wide association study (OR 1.43 [95% CI 1.35–1.51]) [9]. Guo et al. [31] reported that human umbilical vein endothelial cells with the *2 allele produced higher asymmetric dimethylarginine, an endogenous competitive inhibitor of NO synthase, than that of cells with the *1/*1 allele, leading to endothelial dysfunction. *ALDH2* knockout mice displayed exacerbated cardiac damage following ischemia-reperfusion with increased formation of toxic aldehyde radicals [6], Additionally. ALDH2 activator [5] and *ALDH2* overexpression [6] attenuated infarct size in a rodent model of myocardial infarction. The present study identified a possible impact of ALDH2 activity on myocardial infarction in patients with type 2 diabetes mellitus, irrespective of alcohol consumption (Table 4). This finding suggests that cardioprotective effects of moderate alcohol consumption may not be applied to ALDH2 *2 carriers, at least in Japanese patients with type 2 diabetes. In contrast, information regarding the relationship between the *ALDH2* SNP and brain infarction is scarce. One Japanese study revealed that there was no relationship between lacunar infarction by MRI and the *ALDH2* rs671 SNP in the general population [32]. However, ALDH2 activation or overexpression protected the brain against stroke in rats [7]. The present study suggests that ALDH2 deficiency as a result of the ALDH2 *2 allele may be a risk factor for brain infarction development in Japanese patients with type 2 diabetes mellitus. However, since alcohol consumption is linearly associated with stroke risk in a Japanese population, rather than the U-shaped fashion observed in Caucasians [33], Japanese drinkers in general have an increased risk for brain infarction.

The strength of the present study is that we included a relatively large number of participants and collected data on a range of parameters. However, there were some limitations to the present study. The cross-sectional and observational nature did not address causation. However, there have been no large-scale randomized clinical trials using alcoholic beverages because of ethical and practical issues. In addition, alcohol consumption estimated by self-administered questionnaire is often underreported in drinkers with diabetes who should be advised to reduce alcohol intakes. To overcome this problem, using a genetic variant may be useful, because alcohol consumption is strongly linked with *ALDH2* genotype in a Japanese population [4) Recently, a functional *ADHB1* SNP has been used in Caucasians for the same purpose by Mendelian randomization analysis [34]. Finally, this study performed multiple testing; however, the Tukey–Kramer test used in the present study has been widely used for post hoc testing [35].

## Conclusions

The present study revealed that patients with type 2 diabetes with the ALDH2 *2 allele had a lower microvascular complication prevalence associated with alcohol consumption. In contrast, patients displayed a higher macrovascular complication prevalence irrespective of alcohol consumption. Further studies will be required to investigate the contrasting effects of genetically determined ALDH2 activity on microvascular and macrovascular complications in patients with type 2 diabetes mellitus. However, increasing alcohol consumption may not be recommended in ALDH2 *2 carriers with type 2 diabetes mellitus, because accumulated acetaldehyde is known to be a significant carcinogen in humans [36].
